# Small or Non-Small Cell Lung Cancer Based Therapy for Treatment of Large Cell Neuroendocrine Cancer of The Lung? University of Cincinnati Experience

**DOI:** 10.1155/2018/9761826

**Published:** 2018-11-01

**Authors:** Ihab Eldessouki, Ola Gaber, Tariq Namad, Jiang Wang, John C. Morris, Nagla Abdel Karim

**Affiliations:** ^1^Department of Hematology-Oncology, Vontz Center for Molecular Studies, University of Cincinnati, 3125 Eden Ave, Cincinnati, OH 45267, USA; ^2^Department of Pathology, University of Cincinnati, Laboratory Medicine Building, Suite 110, 234 Goodman Street, Cincinnati, OH 45219-0533, USA

## Abstract

Large cell neuroendocrine cancer (LCNEC) of the lung exhibits morphological and immunohistochemical characteristics of both neuroendocrine and large cell carcinomas. No defined optimal therapy has been described for this subset of patients and the question of whether these patients should be treated with non-small cell lung cancer (NSCLC) treatment protocols, according to the National Comprehensive Cancer Network (NCCN) guidelines, or with small cell lung cancer (SCLC) due to histological and clinical similarities is still uncertain. We conducted a retrospective review of patients identified with diagnosis of LCNEC of the lung at the University of Cincinnati Cancer Center from the year 2002 to 2012 to determine which treatment approach resulted in improved outcomes in this rare category of disease. Patients who received chemotherapy whether NSCLC (group A) or SCLC (group B) protocols did not show significant changes in OS (P=0.911). Meanwhile, patients who underwent surgery (group C) had better OS compared to groups A and B (P= 0.027 and 0.024, respectively). This analysis reveals that outcomes for SCLC or NSCLC treatment strategies in LCNEC patients did not result in survival advantages and future research should be addressing it as a separate entity.

## 1. Background

Clinically, LCNEC of the lung resembles SCLC rather than carcinoid tumors. It is characterized by early nodal and distant metastatic spread [1], and presenting symptoms include cough, hemoptysis, chest pain, dyspnea, and weight loss and this mimics other NSCLC and SCLC clinical presentations; also, paraneoplastic syndromes are considered uncommon in LCNEC [2]. These patients have poor prognosis, and the natural history of the disease closely mimics that of SCLC [1, 3]. Molecular profiling reveals heterogeneous pattern that reveals characteristics of both SCLC and NSCLC, with close resemblance to adenocarcinoma. Rekhtman et al. reported results from 45 resected LCNEC tumors which underwent targeted next-generation sequencing of 241 genes [4]. Fifty-six percent of the tumors had NSCLC-like molecular features, such as lack of* RB1*+*TP53* coalteration and presence of NSCLC-type common mutations including* STK11, KRAS, KEAP1, and NFE2L2*, while 40% showed SCLC-like molecular features, with* RB1*+*TP53* coalteration, complete absence of mutations* in STK11* and* KRAS*, and exclusive or enriched occurrence of* MYCL, SOX2,* and* FGFR1* amplifications and mutation and/or loss* of PTEN *[4]. So far, no standard treatment approach for LCNEC has been defined and existing recommendations are extrapolation of NSCLC and SCLC therapies. The reasons may include rarity of the disease, but the lack of randomized prospective trials is a major factor. [3]. According to the NCCN guidelines, LCNEC can be managed as per NSCLC guidelines; however, others prefer same chemotherapeutic regimens for SCLC, based on the fact that both are high-grade neuroendocrine tumors and due to their similar clinical course [5]. Even though resection is recommended for early stage LCNEC [6], the expected prognosis remains poor with a 5-year survival rate of 27 to 67% [7]. The role of adjuvant chemotherapy is only supported by data obtained from small prospective phase II studies [8] and retrospective experiences with adjuvant treatment [9, 10]. SCLC regimens (platinum + etoposide) are suggested to be more effective and beneficial than those of NSCLC [11].

Another debatable aspect in LCNEC management is radiotherapy and prophylactic cranial irradiation (PCI) in early stages. Despite the poorer prognosis and short survival, available evidence supports administration of radiotherapy according to NSCLC guidelines. The low incidence of spontaneous brain metastases (about 25%) does not support routine PCI as in SCLC [12].

In advanced stage LCNEC, SCLC regimens more commonly practiced which is based on limited retrospective analyses [13] and according to the recommendations of American Society of Clinical Oncology (ASCO) in 2015 [14]. Recently, it was suggested that there is an increase in the OS in LCNEC patients with NSCLC regimens which are adopted, specially gemcitabine-platinum rather than pemetrexed-platinum and etoposide-platinum (SCLC-based) regimens [15]. In this study, we are reporting the results of a retrospective analysis of LCNEC patients treated in the University of Cincinnati medical center from 2002 to 2012. Our aim was to see how the treatment approach affected survival, responses, and prognosis of pulmonary LCNEC.

## 2. Methods

A retrospective review of patients identified with a pathological diagnosis of LCNEC of the lung. Database of Medical Center of the University of Cincinnati was searched from the year 2002 to 2012, and all patients with LCNEC histology were identified. Patients with incomplete records were removed from the study.

Paraffin blocks were retrieved from the tissue bank for the eligible subjects and their diagnosis was reviewed according to WHO 2015 guidelines. Tissue markers tested included CD56, synaptophysin, chromogranin A, and Ki-67 [2, 16–18].

Survival probabilities were estimated by Kaplan Meier method and differences in survival were compared by the log-rank test. Uni- and multivariable predictors of overall mortality were estimated by Cox-regression analysis. Overall survival was defined as the time from diagnosis till the time of death from any cause.

## 3. Results

### 3.1. Demographics

Searching the database of the Cancer Center of the University of Cincinnati, we were able to identify 26 patients diagnosed as LCNEC of the lung. The median age of the patients was 58 years (range: 42-77 years), and males to females ratio was 1.2. Six patients (23.1%) were nonsmokers, and 14 patients (53.8%) were heavy smokers. For patients' demographics, refer to Table 1.

### 3.2. Patient Management

Nine patients (34.6%) were treated as SCLC patients, i.e., received cisplatin/etoposide for their initial treatment, and 6 patients (23.1%) received a NSCLC-based treatment. The median OS for all patients was 16 months (range: 11 to 42 months), and OS of patients treated with chemotherapy was found to be statistically insignificant compared to those who never received chemotherapy (HR 0.43 and 0.79; p=0.23 and 0.71). Patients who underwent surgery had a higher risk of death (HR of 3.66; p=0.04).

### 3.3. Mortality Hazard and Survival Analysis

In the univariate model analysis, increasing age was found to be statistically significant (HR: 0.95, 95% CI: 0.90-0.99, P = 0.032), and group C patients never received chemotherapy during their course of treatment, and surgery was their main modality of treatment (HR 0.26, 95% CI: 0.08-0.87, P = 0.029). On multivariable analysis (MVA), group C was associated with the highest statistical significance (HR: 0.30, 95% CI: 0.05-1.81, P = 0.190). The stage III/IV disease overall mortality was higher than stages I/II; however this did not represent statistical significance (HR:2.26, P=0.128); see Table 2.

### 3.4. Kaplan-Meier Survival Analysis

**Figure 1:**
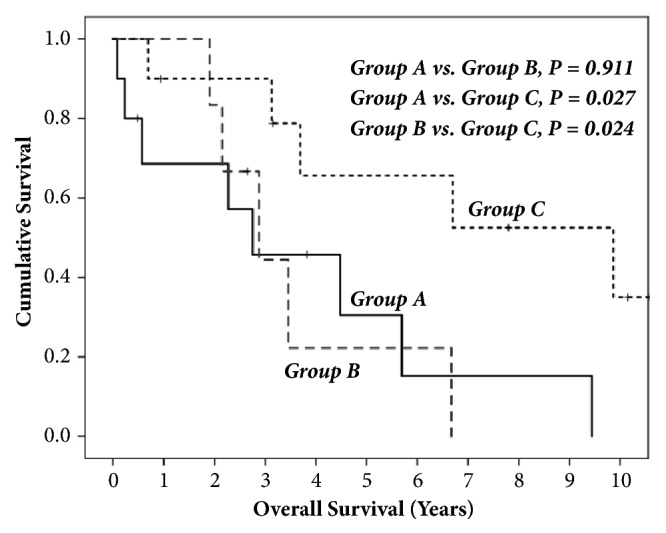
The figure compares the OS in years against the cumulative survival of each treatment group. Groups A and B compared did not have statistical significance (p=0.911). Comparing groups A and B to group C, the latter exhibits significance in survival with both groups (p=0.027 and 0.024, respectively).

Median survival (95% CI) for each treatment group of A, B, and C was 2.75 (0.05-5.45), 2.88 (1.46-4.30), and 9.87 years (2.56-17.18), respectively. Group C patients had the best overall survival (OS) rate [5-year OS of 65.6% and median survival of 9.87 years (95% CI: 2.56-17.18)], compared to those who received group A chemotherapy regimen [5-year OS of 30.5% and median survival of 2.75 years (95% CI: 0.05-5.45)] or to those who received group B chemotherapy regimen [5-year OS of 22.2% and median survival of 2.88 years (95% CI: 1.46-4.30)][Figure 1].

## 4. Discussion

Molecular and genetic analysis of lung cancers have enabled defining subcategories that are now the foundation for targeted and immunotherapy. However, rare and ambiguous subtypes such as LCNEC of the lung and primary pulmonary sarcomatoid carcinoma (PPSC) are underpresented, and their treatment strategies are still based on more common lung tumor histologies such as NSCLC though they represent clinically and histologically independent entities [19–21]. In a study performed on 45 LCNEC patients by Sun et al., the authors reported that the response rates were 73% and 50% for SCLC and NSCLC treatment-based groups (P = 0.19), respectively. The median progression free survival was higher for the SCLC group (6.1 versus 4.9 months, P = 0.41), and the OS showed that SCLC group was also at advantage (16.5 versus 9.2 months, P = 0.10) compared to NSCLC-based treated group [13]. Our results showed that the NSCLC group did not show a survival advantage compared to the SCLC group. Although NSCLC group had slightly higher median OS (0.05 years) than SCLC treatment-based group, the 5-year OS was lower in comparison; however, there was no statistical significance in both results (p=0.911). LCNEC of the lung prognostic factors was reported to be similar to SCLC rather than carcinoid tumors [1]. In a study comparing the 5-year survival of stage I LCNEC and poorly differentiated NSCLC patients, significant higher survival rates were revealed in the poorly differentiated NSCLC patients (67% versus 88%, p=003) [22], indicating that poor effect of currently executed regimens for these rare but rather specific categories. A retrospective analysis revealed that LCNEC 5-year survival rates range from 15 to 57%, and diagnosis at an early stage showed slight improvement in these cases (27–67 %) [23], regardless of treatment strategy. Stage to stage 5-year survival comparison of pulmonary LCNEC and NSCLC was 54.5% versus 89.3% for the adenocarcinoma or squamous cell carcinoma patients [24]. It is common for LCNEC of the lung to metastasize to distant sites [22], and adjuvant treatment was suggested in most platforms [13, 23, 25, 26]. However reports were more in favor of platinum based therapies with unsatisfactory outcomes (recurrence rate =46%) [22], even though platinum based regimens yielded higher response rates compared to NSCLC [13].

## 5. Conclusions

LCNEC of the lung has poor prognosis and survival. Studies report inconsistencies in the results from these patients' treatment. Even though group C patients, who were treated with surgery only, had better survival, recurrences were common and survival was poor. More randomized trials are needed to address this category of tumors probably based on tumor specific targeted treatment. Programmed cell death 1 (PD-1)/programmed cell death ligand 1 (PD-L1) expression, immunotherapy, and their outcomes in LCNEC open a new door for considering a new standard treatment [27–31].

## Figures and Tables

**Table 1:** Showing the demographics of 26 patients diagnosed with LCNEC of the lung.

Patients	Age (years)	Race	Gender	Smoking Status (pack/year)	Traetment
1	54	AA	M	34	SCLC
2	59	AA	F	Non-smoker	SCLC
3	61	W	F	43	SCLC
4	56	W	F	36	SCLC
5	51	AA	F	30	-
6	76	AA	M	32	-
7	78	W	F	Non-smoker	-
8	73	AA	F	46	-
9	69	W	M	33	-
10	58	W	M	Non-smoker	-
11	63	W	F	Non-smoker	NSCLC
12	64	AA	M	Non-smoker	-
13	59	AA	M	15	NSCLC
14	49	AA	F	18	NSCLC
15	64	W	F	44	-
16	59	AA	M	15	SCLC
17	49	W	M	18	NSCLC
18	54	W	M	Non-smoker	SCLC
19	64	W	M	50	-
20	47	AA	F	20	-
21	56	W	M	40	SCLC
22	60	AA	F	4	-
23	61	W	F	47	NSCLC
24	44	AA	M	26	NSCLC
25	59	W	M	24	-
26	56	W	M	60	SCLC

**Table 2:** Showing analysis of overall mortality for 26 patients of LCNEC of the lung.

**Cox-regression analysis of overall mortality (events =18/26)**	**Univariate analysis**				**Multivariable analysis** **∗**			
	**HR**	**95**%** CI**		**P value**	**HR**	**95**%** CI**		**P value**
		**Lower**	**Upper**			**Lower**	**Upper**	
Age	0.95	0.90	0.99	***0.032***	0.99	0.92	1.07	0.797
Gender								
Female	Reference							
Male	1.19	0.47	3.03	0.720				
Race								
White	Reference							
African American	1.50	0.59	3.81	0.394				
Smoking History								
Non-Smoker	Reference							
Former/Current Smoker	0.81	0.29	2.28	0.688				
Stage								
Stages I/II	Reference							
Stages III/IV	2.26	0.75	6.43	0.128				
Chemotherapy Regimen								
A	Reference				Reference			
B	1.12	0.36	3.53	0.847	1.09	0.34	3.50	0.882
C	0.26	0.08	0.87	***0.029***	0.30	0.05	1.81	***0.190***
*∗Univariate variables with P value <0.1 were included in the multivariable model.*								

A: SCLC chemotherapy, B: NSCLC chemotherapy, C: no chemotherapy (surgical), and HR: hazard ratio.

## Data Availability

The data used to support the findings of this study are included within the article.
